# Supplementary material for: Potential Use of Vacuum Impregnation and High-Pressure Homogenization to Obtain Functional Products from Lulo Fruit (Solanum quitoense Lam.)
Source: Foods. 2021 Apr 9;10(4):817. doi: 10.3390/foods10040817 (PMC8069265; doi:10.3390/foods10040817)
Supplement: Supplementary file 1 [file foods-10-00817-s001.zip › Suplementari files/Table S3. Comp. File.docx]

Table S3. Phytochemical profile of homogenized at 50MPa lulo juice by high resolution LC-MS /MS.

| **Homogenized 50 MPa** | | | | | | |
| --- | --- | --- | --- | --- | --- | --- |
| **Compound name** | **RT (min)** | **M/Z experimental** | **Teoric mass** | **MS/MS fragments** | **Molecular Formula** | **Error (ppm)** |
| **Hydroxycinnamic acids** [**[M − H] ¯** |  |  |  |  |  |  |
| 3-Caffeoylquinic acid | 12.76 | 353.0878 | 353.0875 | 190/84 | C_16_H_18_O_9_ | -0.8 |
| 5-Caffeoylquinic acid | 12.76 | 353.0875 | 353.0878 | 190/84 | C_16_H_18_O_9_ | -0.8 |
| 1-Caffeoylquinic acid | 12.76 | 353.0875 | 353.0878 | 190/84 | C_16_H_18_O_9_ | -0.8 |
| Cis-3-Caffeoylquinic acid | 12.76 | 353.0875 | 353.0878 | 190/84 | C_16_H_18_O_9_ | -0.8 |
| Cis-5-Caffeoylquinic acid | 12.76 | 353.0875 | 353.0878 | 190/84 | C_16_H_18_O_9_ | -0.8 |
| Trans-5-Caffeoylquinic acid | 12.76 | 353.0875 | 353.0878 | 190/84 | C_16_H_18_O_9_ | -0.8 |
| Trans-3-Caffeoylquinic acid | 12.67 | 353.0875 | 353.0878 | 190/84 | C_16_H_18_O_9_ | -0.8 |
| p-Coumaric acid 4-O-glucoside | 12.8 | 325.0929 | 325.0928 | 144/118/116 | C_15_H_18_O_8_ | -0.2 |
| p-Coumaroyl glucose | 12.95 | 325.093 | 325.0929 | 199/144/142//116 | C_15_H_18_O_8_ | 0.5 |
| Caffeic acid | 13.13 | 179.0351 | 179.035 | 134/133/107/88 | C_9_H_8_O_4_ | 0.7 |
| Cis-Caffeic acid | 13.13 | 179.0351 | 179.035 | 134/133/107/88 | C_9_H_8_O_4_ | 0.7 |
| Trans-Caffeic acid | 13.13 | 179.0351 | 179.035 | 134/133/107/88 | C_9_H_8_O_4_ | 0.7 |
| Ferulic acid 4-O-glucoside | 13.38 | 355.1035 | 355.103 | 174/159/131 | C_16_H_20_O_9_ | -1.3 |
| Feruloyl glucose | 13.38 | 355.1035 | 355.103 | 174/159/131 | C_16_H_20_O_9_ | -1.3 |
| 5-Feruloylquinic acid | 14.84 | 367.1035 | 367.1031 | - | C_17_H_20_O_9_ | -0.9 |
| 4-Feruloylquinic acid | 14.84 | 367.1035 | 367.1031 | - | C_17_H_20_O_9_ | -0.9 |
| 3-Feruloylquinic acid | 14.84 | 367.1031 | 367.1035 | - | C_17_H_20_O_9_ | -0.9 |
| **Flavonoids [M − H] ¯** | | | | | | |
| Quercetin 3-O-rhamnosyl-galactoside | 15.8 | 609.1467 | 609.1461 | 299/270 | C_27_H_30_O_16_ | 1 |
| Quercetin 3-O-rhamnosyl-galactoside | 15.8 | 609.1467 | 609.1461 | 299/270 | C_27_H_30_O_16_ | 1 |
| Kaempferol 3,7-O-diglucoside | 15.8 | 609.1467 | 609.1461 | 299/270 | C_27_H_30_O_16_ | 1 |
| Kaempferol 3-O-sophoroside | 15.8 | 609.1461 | 609.1467 | 299/270 | C_27_H_30_O_16_ | 1 |
| Quercetin 3-O-galactoside 7-O-rhamnoside | 15.8 | 609.1467 | 609.1461 | 299/270 | C_27_H_30_O_16_ | 1 |
| *Table x (continued)* |  |  |  |  |  |  |
| **Compound name** | **RT (min)** | **M/Z experimental** | **Teoric mass** | **MS/MS fragments** | **Molecular fornula** | **Error (ppm)** |
| Isorhamnetin 3-O-glucoside 7-O-rhamnoside | 16.36 | 623.1618 | 623.162 | 313/298/270 | C_28_H_32_O_16_ | 0.4 |
| Kaempferol 3-O-galactoside 7-O-rhamnoside | 16.4 | 593.1512 | 593.1512 | 283/254 | C_27_H_30_O_15_ | 0 |
| Kaempferol 3-O-rutinoside | 16.4 | 593.1512 | 593.1512 | 283/254 | C_27_H_30_O_15_ | 0 |
| Kaempferol 3-O-galactoside | 17.3 | 447.0933 | 447.0929 | - | C_21_H_20_O_11_ | -0.9 |
| Kaempferol 3-O-glucoside | 17.3 | 447.0933 | 447.0929 | - | C_21_H_20_O_11_ | -0.9 |
| Kaempferol 7-O-glucoside | 17.3 | 447.0929 | 447.0933 | - | C_21_H_20_O_11_ | -0.9 |
| Quercetin 3-O-rhamnoside | 17.3 | 447.0933 | 447.0929 | - | C_21_H_20_O_11_ | -0.9 |
| 3,7-Dimethylquercetin | 18.79 | 329.0666 | 329.0667 | - | C_17_H_14_O_7_ | -0.2 |
| **Phenolic acids [M − H] ¯** | | | | | | |
| 2-Hydroxybenzoic acid | 11.25 | 137.0245 | 137.0244 | 107 | C_7_H_6_O_3_ | 0.9 |
| 4-Hydroxybenzoic acid | 11.25 | 137.0245 | 137.0244 | 107 | C_7_H_6_O_3_ | 0.9 |
| Sesamol | 11.25 | 137.0244 | 137.0245 | 107 | C_7_H_6_O_3_ | 0.9 |
| 2-Hydroxybenzoic acid | 11.25 | 137.0245 | 137.0244 | 107 | C_7_H_6_O_3_ | 0.9 |
| 4-Hydroxybenzoic acid 4-O-glucoside | 12.67 | 299.0774 | 299.0772 | 92/136 | C_13_H_16_O_8_ | 0.5 |
| Benzoic acid | 14.06 | 121.0296 | 121.0295 | 91 | C_7_H_6_O_2_ | 0.7 |
| 4-Methoxybenzoic acid | 15.04 | 151.0401 | 151.04 | 90 | C_8_H_8_O_3_ | -0.2 |
| **Other phenolics [M − H] ¯** | | | | | | |
| Protocatechuic aldehyde | 11.25 | 137.0245 | 137.0244 | 107 | C_7_H_6_O_3_ | 0.9 |
| Coumarin | 12.94 | 145.0296 | 145.0295 | 116/115/87 | C_9_H_6_O_2_ | 0.6 |
| 3,4-Dihydroxyphenyl-2-oxypropanoic acid | 13.13 | 179.035 | 179.0351 | 134/108/88 | C_9_H_8_O_4_ | 0.7 |
| 4-Hydroxybenzaldehyde | 14.06 | 121.0295 | 121.0296 | 91 | C_7_H_6_O_2_ | 0.7 |
| Chrysoeriol 7-O-apiosyl-glucoside | 16.4 | 593.1512 | 593.1512 | 254/283 | C_27_H_30_O_15_ | 0 |
| Homovanillyl alcohol | 18.1 | 167.0715 | 167.0714 | - | C_9_H_12_O_3_ | 0.8 |
| Jaceosidin | 18.79 | 329.0667 | 329.0666 | - | C_17_H_14_O_7_ | 0 |
| Neoeriocitrin  *Table (Contined)* | 16.15 | 595.1672 | 595.1668 | 270/234/192/134/106 | C_27_H_32_O_15_ | 0.5 |
| **Compound name** | **RT (min)** | **M/Z experimental** | **Teoric mass** | **MS/MS fragments** | **Molecular fornula** | **Error (ppm)** |
| Narirutin 4-O-glucoside | 16.82 | 301.0716 | 301.0718 | - | C_16_H_14_O_6_ | -0.4 |
| Hesperetin | 16.82 | 301.0716 | 301.0718 | - | C_16_H_14_O_6_ | -0.4 |
| Hesperidin | 16.8 | 609.1829 | 609.1825 | 300/150 | C_28_H_34_O_15_ | 0.7 |
| **Flavones [M − H] ¯** |  |  |  |  |  |  |
| Apigenin 6,8-di-C-glucoside | 16.4 | 593.1512 | 593.1512 | 283/254 | C_27_H3_0_O_15_ | 0 |
| Luteolin 4-O-glucoside | 17.3 | 447.0933 | 447.0929 | - | C_21_H_20_O_11_ | -0.9 |
| 6-Hydroxyluteolin 7-O-rhamnoside | 17.3 | 447.0929 | 447.0933 | - | C_21_H_20_O_11_ | -0.9 |
| **Dhydrochalcones** **[M − H] ¯** | | | | | | |
| Phloridzin | 17.21 | 435.1293 | 435.1297 | 272/188/178/166/150/112/92 | C_21_H_24_O_10_ | -0.9 |
