# Supplementary material for: Potential Use of Vacuum Impregnation and High-Pressure Homogenization to Obtain Functional Products from Lulo Fruit (Solanum quitoense Lam.)
Source: Foods. 2021 Apr 9;10(4):817. doi: 10.3390/foods10040817 (PMC8069265; doi:10.3390/foods10040817)
Supplement: Supplementary file 1 [file foods-10-00817-s001.zip › Suplementari files/Table S2. Comp. File.docx]

Table S2. Phytochemical profile of non- homogenized lulo juice by high resolution LC-MS/MS

| **Non-homogenized** | | | | | | | | |
| --- | --- | --- | --- | --- | --- | --- | --- | --- |
| **Compound name** | **RT (min)** | **M/Z experimental** | **Teoric mass** | **MS/MS fragments** | | **Molecular Formula** | | **Error (ppm)** |
| **Hydroxycinnamic acids [M − H] ¯** | | | | | | | | |
| Caffeic acid | 11.13 | 179.035 | 179.035 | 134/133/88 | | C_9_H_8_O_4_ | | 0.1 |
| Cis-Caffeic acid | 13.66 | 179.035 | 179.035 | 134/133/88 | | C_9_H_8_O_4_ | | 0.1 |
| Trans-Caffeic acid | 13.66 | 179.035 | 179.035 | 134/133/88 | | C_9_H_8_O_4_ | | 0.1 |
| 3-Caffeoylquinic acid | 13.29 | 353.0886 | 353.0878 | 191/179 | | C_16_H_18_O_9_ | | 2.4 |
| 4-Caffeoylquinic acid | 13.29 | 353.0886 | 353.0878 | 190/84 | | C_16_H_18_O_9_ | | 2.4 |
| 1-Caffeoylquinic acid | 13.29 | 353.0878 | 353.0886 | 190 | | C_16_H_18_O_9_ | | 2.4 |
| Cis-3-Caffeoylquinic acid | 13.29 | 353.0886 | 353.0878 | 190/84 | | C_16_H_18_O^9^ | | 2.4 |
| Trans-5-Caffeoylquinic acid | 13.29 | 353.0886 | 353.0878 | 190/84 | | C_16_H_18_O_9_ | | 2.4 |
| Cis-5-Caffeoylquinic acid | 13.29 | 353.0878 | 353.0886 | 190/84 | | C_16_H_18_O_9_ | | 2.4 |
| Trans-3-Caffeoylquinic acid | 13.29 | 353.0878 | 353.0886 | 190/84 | | C_16_H_18_O_9_ | | 2.4 |
| 5-Caffeoylquinic acid | 13.29 | 353.0886 | 353.0878 | 190/84 | | C_16_H_18_O_9_ | | 2.4 |
| 5-Feruloylquinic .acid | 15.43 | 367.1035 | 367.104 | 190/133/92 | | C_17_H_20_O_9_ | | 1.4 |
| 3-Feruloylquinic acid | 15.43 | 367.1035 | 367.104 | 190/133/92 | | C_17_H_20_O_9_ | | 1.4 |
| 4-Feruloylquinic acid | 15.43 | 367.1040 | 367.1035 | 190/133/92 | | C_17_H_20_O_9_ | | 1.4 |
| Feruloyl glucose | 13.93 | 355.1037 | 355.1035 | 174/159/131 | | C_16_H_20_O_9_ | | 0.8 |
| p-Coumaroylquinic acid | 15.06 | 337.0932 | 337.0929 | 190/172/118/92 | | C_16_H_18_O_8_ | | 0.6 |
| 3-p-Coumaroylquinic acid | 15.06 | 337.0932 | 337.0929 | 190/172/118/92 | | C_16_H_18_O_8_ | | 1 |
| 4-p-Coumaroylquinic acid | 15.06 | 337.0932 | 337.0929 | 190/172/118/92 | | C_16_H_18_O_8_ | | 1 |
| 5-p-Coumaroylquinic acid | 15.06 | 337.0932 | 337.0929 | 190/172/118/92 | | C_16_H_18_O_8_ | | 1 |
| **Phenolic acids [M − H] ¯** | | | | | | | | |
| 3-Hydroxybenzoic acid | 11.73 | 137.0244 | 137.0244 | - | | C_7_H_6_O_3_ | | 0.1 |
| 4-Hydroxybenzoic acid | 11.73 | 137.0244 | 137.0244 | - | | C_7_H_6_O_3_ | | 0.1 |
| Benzoic acid | 14.67 | 121.0295 | 121.0296 | 91 | | C_7_H_6_O_2_ | | 0.9 |
| 4-Methoxybenzoic acid | 15.62 | 151.0402 | 151.0401 | n.d | | C_8_H_8_O_3_ | | 0.6 |
| *Table x (continued)* |  |  |  |  |  | |  | |
| **Compound name** | **RT (min)** | **M/Z experimental** | **Teoric mass** | **MS/MS fragments** | **Molecular fornula** | | **Error (ppm)** | |
| **Other phenolics** **[M − H] ¯** | | | | | | | | |
| Protocatechuic aldehyde | 11.73 | 137.0244 | 137.0244 | - | | C_7_H_6_O_3_ | | 0.1 |
| Sesamol | 11.73 | 137.0244 | 137.0244 | - | | C_7_H_6_O_3_ | | 0.1 |
| 4-Hydroxycoumarin | 13.23 | 161.0244 | 161.0245 | 143/131/103/88 | | C_9_H_6_O_3_ | | 0.8 |
| Umbelliferone | 13.23 | 161.0244 | 161.0245 | 143/131/103/88 | | C_9_H_6_O_3_ | | 0.1 |
| 3,4-Dihydroxyphenyl-2-oxypropanoic acid | 13.66 | 179.035 | 179.035 | 134/133/88 | | C_33_H_42_O_19_ | | 3.4 |
| 4-Hydroxybenzaldehyde | 14.67 | 121.0295 | 121.0296 | - | | C_7_H_6_O_2_ | | 0.9 |
| 1-Sinapoyl-2-feruloylgentiobiose | 16.12 | 723.2142 | 723.2177 | 542/366/190 | | C_33_H_40_O_18_ | | 4.8 |
| Chalconaringenin | 18.8 | 271.0612 | 271.0619 | 82/116/118 | | C_15_H_12_O_5_ | | 2.6 |
| Coumarin | 13.32 | 145.0295 | 145.0295 | 106/98/88 | | C_9_H_6_O_2_ | | 0.2 |
| p-Coumaric acid 4-O-glucoside | 13.31 | 325.0941 | 325.0929 | 144//116/118 | | C_15_H_18_O_8_ | | 3.7 |
| p-Coumaroyl glucose | 13.31 | 325.0941 | 325.0929 | 144/119/116 | | C_15_H_18_O_9_ | | 3.7 |
| **Flavanones** **[M − H] ¯** | | | | | | | | |
| Narirutin 4-O-glucoside | 14.21 | 741.2248 | 741.2263 | 208/193 | | C_33_H_42_O_19_ | | 2.1 |
| Pelargonidin 3,5-O-diglucoside | 16.82 | 594.1579 | 594.159 | - | | C_27_H_31_O_15_ | | -1.8 |
| Narirutin | 17.09 | 579.1719 | 579.1743 | 270/150 | | C_27_H_32_O_14_ | | 4.1 |
| Naringin | 17.09 | 579.1719 | 579.1743 | 270/150 | | C_27_H_32_O_14_ | | 4.1 |
| Engeletin | 17.78 | 433.1147 | 433.114 | 270/226/150/118/106/82 | | C_21_H_22_O_10_ | | 1.5 |
| Naringenin 7-O-glucoside | 17.78 | 433.1147 | 433.114 | 226/150/118/106/82 | | C21H22O10 | | 1.5 |
| Naringenin | 18.8 | 271.0612 | 271.0619 | 118/116/82 | | C_15_H_12_O_5_ | | 2.6 |
| Butein | 18.8 | 271.0612 | 271.0619 | 186/150/118/116/82 | | C_15_H_12_O_5_ | | 2.6 |
| **Anthocynins [M − H] ¯** | | | | | | | | |
| Cyanidin 3-O-rutinoside | 16.82 | 594.159 | 594.1579 | - | | C_27_H_31_O_15_ | | -1.8 |
| Pelargonidin 3,5-O-diglucoside | 16.82 | 594.1579 | 594.159 | - | | C_27_H_31_O_15_ | | -1.8 |
