# Supplementary material for: Potential Use of Vacuum Impregnation and High-Pressure Homogenization to Obtain Functional Products from Lulo Fruit (Solanum quitoense Lam.)
Source: Foods. 2021 Apr 9;10(4):817. doi: 10.3390/foods10040817 (PMC8069265; doi:10.3390/foods10040817)
Supplement: Supplementary file 1 [file foods-10-00817-s001.zip › Suplementari files/Table S6. Comp. File.docx]

Table S7. Polyamines identified in lulo fruit and its non-homogenized and homogenized juice at different homogenization pressures by high performance LC-MS/MS.

| **Compound** | **Sample** | **Rt (Min)** | **[M-H]- / [M-H]+** | MS^2^ fragments m/z | **Molecular Formula** |
| --- | --- | --- | --- | --- | --- |
| N, N´-bis(dihydrocaffeoyl) spermine | LF, NHJ, H50J, H100J, H150J | 13.12 | 472/474 | 350 (40),308 (40) | C_25_H_35_N_3_O_6_ |
| N, N´N-tris(dihydrocaffeoyl) spermine |  | 16.4 | 637/638 | 514(25), 472(30) | C_34_H_43_N_3_O_9_ |
| N, N´-bis(dihydrocaffeoyl) spermine hexoside |  | 16.6 | 653/653 | - | C_31_H_47_N_3_O_12_ |
| N, N, N´-tris(dihydrocaffeoyl) spermine hexoside |  | 17.8 | 817/818 | - | C4_0_H_55_N_3_O_15_ |
| N, N´-bis(dihydrocaffeoyl) spermine dihexoside |  | 18.9 | 833/834 | - | C_37_H_59_N_3_O_18_ |

LF: Lulo fruit; NHJ: non-homogenized juice; H50J: homogenized lulo juice at 50 MPa ; H100J: homogenized lulo juice at 100MPa; H150J: homogenized lulo juice at 150MPa. Confirmed with MS fragmentation and database results; Confirmed with reference
