# Supplementary material for: Potential Use of Vacuum Impregnation and High-Pressure Homogenization to Obtain Functional Products from Lulo Fruit (Solanum quitoense Lam.)
Source: Foods. 2021 Apr 9;10(4):817. doi: 10.3390/foods10040817 (PMC8069265; doi:10.3390/foods10040817)
Supplement: Supplementary file 1 [file foods-10-00817-s001.zip › Suplementari files/Table S1. Comp. File.docx]

Table S1. Phytochemical profile of the lulo fruit extract by high resolution LC-MS /MS.

| **Lulo fruit** | | | | | | |
| --- | --- | --- | --- | --- | --- | --- |
| **Compound name** | **RT (min)** | **M/Z experimental** | **Teoric mass** | **MS/MS fragments** | **Molecular Formula** | **Error (ppm)** |
| **Hydroxycinnamic acids** [M−H]¯ | | | | | | |
| Caffeic acid | 11.13 | 179.035 | 179.035 | 134/133/88 | C_9_H_8_O_4_ | 0.1 |
| 3-Caffeoylquinic acid | 13.15 | 353.0881 | 353.0878 | 191/179 | C^16^H_18_O_9_ | 0.8 |
| 4-Caffeoylquinic acid | 13.15 | 353.0881 | 353.0878 | - | C^16^H_18_O_9_ | 0.8 |
| 5-Caffeoylquinic acid | 13.15 | 353.0881 | 353.0878 | 191 | C^16^H_18_O_9_ | 0.8 |
| Trans-3-Caffeoylquinic acid | 13.15 | 353.0881 | 353.0878 | - | C^16^H_18_O_9_ | 0.8 |
| 1-Caffeoylquinic acid | 13.15 | 353.0881 | 353.0878 | - | C^16^H_18_O_9_ | 0.8 |
| Cis-3-Caffeoylquinic acid | 13.15 | 353.0881 | 353.0878 | - | C^16^H_18_O_9_ | 0.8 |
| Trans-5-Caffeoylquinic acid | 13.15 | 353.0881 | 353.0878 | 191/179 | C^16^H_18_O_9_ | 0.8 |
| Cis-5-Caffeoylquinic acid | 13.15 | 353.0881 | 353.0878 | 191/179 | C^16^H_18_O_9_ | 0.8 |
| Ferulic acid 4-O-glucoside | 13.91 | 355.1038 | 355.1035 | 131/159/174 | C^16^H_18_O_9_ | 1 |
| Feruloyl glucose | 13.91 | 355.1038 | 355.1035 | 131/159/174 | C^16^H_18_O_9_ | 1 |
| Sinapic acid | 13.91 | 223.0613 | 223.0612 | 92/120 | C_11_H_12_O_5_ | 0.6 |
| Sinapoyl glucose | 13.92 | 385.1144 | 385.114 | 174/189 | C_1_7H_22_O_10_ | 0.9 |
| Sitostanyl ferulate | 20.8 | 607.4377 | 607.4368 | 265 | C_39_H_60_O_5_ | 1.5 |
| **Flavonoids** [M−H] | | | | | | |
| Quercetin 3,4-O-diglucoside | 15.74 | 625.1432 | 625.141 | 270/299 | C_27_H_30_O_17_ | 3.5 |
| Quercetin 3-O-glucosyl-glucoside | 15.74 | 625.1432 | 625.141 | 270/299 | C_27_H_30_O_17_ | 3.5 |
| Quercetin 3-O-sophoroside | 15.74 | 625.1432 | 625.141 | 299 | C_27_H_30_O_17_ | 3.5 |
| Myricetin 3-O-rutinoside | 15.74 | 625.1432 | 625.141 | 299/301 | C_27_H_30_O_17_ | 3.5 |
| Kaempferol 3,7-O-diglucoside | 16.11 | 609.1491 | 609.1461 | 270/299 | C_27_H_30_O_16_ | 4.8 |
| Kaempferol 3-O-sophoroside | 16.11 | 609.1491 | 609.1461 | 299 | C_27_H_30_O_16_ | 4.8 |
| Quercetin 3-O-rhamnosyl-galactoside | 16.11 | 609.1491 | 609.1461 | 299 | C_27_H_30_O_16_ | 4.8 |
| *Table xxxxx (continued)* | | | | | | |
| **Compound name** | **RT (min)** | **M/Z Experimental** | **Teoric mass** | **MS/MS fragments** | **Molecular Formula** | **Error (ppm)** |
| Quercetin 3-O-galactoside 7-O-rhamnoside | 16.11 | 609.1491 | 609.1461 | 229 | C_27_H_30_O_16_ | 4.8 |
| Quercetin 3-O-rutinoside | 16.11 | 609.1491 | 609.1461 | 299 | C_27_H_30_O_16_ | 4.8 |
| Kaempferol 3-O-galactoside 7-O-rhamnoside | 16.66 | 593.1536 | 593.1512 | 285 | C_27_H_30_O_15_ | 4.1 |
| Kaempferol 3-O-rutinoside | 16.66 | 593.1536 | 593.1512 | 285 | C_27_H_30_O_15_ | 4.1 |
| Isorhamnetin 3-O-glucoside 7-O-rhamnoside | 16.63 | 623.1639 | 623.1618 | 298/313 | C_28_H_32_O_16_ | 3.4 |
| Isorhamnetin 3-O-rutinoside | 16.63 | 623.1639 | 623.1618 | 298/313 | C_28_H_32_O_16_ | 3.4 |
| Kaempferol 3-O-acetyl-glucoside | 17.86 | 489.1053 | 489.1039 | 254/283 | C_23_H_22_O_12_ | 3.1 |
| Kaempferol 3-O-galactoside | 17.65 | 447.0939 | 447.0933 | 226/254/283 | C_21_H_20_O_11_ | 1.3 |
| Kaempferol 3-O-glucoside | 17.65 | 447.0939 | 447.0933 | 226/254/283 | C_21_H_20_O_11_ | 1.3 |
| Kaempferol 7-O-glucoside | 17.65 | 447.0933 | 447.0939 | 226/254/283 | C_21_H_20_O_11_ | 1.3 |
| Quercetin 3-O-rhamnoside | 17.65 | 447.0939 | 447.0933 | 226/254/283 | C_21_H2_0_O_11_ | 1.3 |
| Isorhamnetin 3-O-glucoside | 17.67 | 477.1049 | 477.1039 | 242/270/284/313 | C_22_H_22_O_12_ | 2.2 |
| Isorhamnetin 4-O-glucoside | 17.67 | 477.1049 | 477.1039 | 242/270/284/313 | C_22_H_22_O_12_ | 2.2 |
| Isorhamnetin 3-O-galactoside | 17.67 | 477.1049 | 477.1039 | 242/270/284/313 | C_22_H_22_O_12_ | 2.2 |
| Kaempferide | 20.34 | 299.0563 | 299.0561 | 88/118/134/184/186 | C_16_H_12_O_6_ | 0.6 |
| **Phenolic acids** [M−H]¯ | | | | | | |
| Benzoic acid | 14.57 | 121.0294 | 121.0295 | - | C_7_H_6_O_2_ | -0.7 |
| **Other phenolics** [M−H]¯ | | | | | | |
| 4-Hydroxybenzaldehyde | 14.57 | 121.0294 | 121.0295 | - | C_7_H_6_O_2_ | -0.7 |
| Chrysoeriol 7-O-apiosyl-glucoside | 16.66 | 593.1536 | 593.1512 | 254/284 | C_27_H_30_O_15_ | 4.1 |
| 6-Hydroxyluteolin 7-O-rhamnoside | 17.65 | 447.0939 | 447.0933 | 226/254/283 | C_21_H_20_O_11_ | 1.3 |
| 4-Vinylphenol | 18.81 | 119.0503 | 119.0502 | 87/88/92 | C_8_H_8_O | 0.7 |
| Eriodictyol | 18.3 | 287.0568 | 287.0561 | 134 | C_15_H_12_O_6_ | 2.3 |
| Chrysoeriol | 20.34 | 299.0563 | 299.0561 | 88/118/184/186 | C_16_H_12_O_6_ | 0.6 |
| Diosmetin | 20.34 | 299.0563 | 299.0561 | 88/118/184/186 | C_16_H_12_O_6_ | 0.6 |
| *Table x (continued)* |  |  |  |  |  |  |
| **Compound name** | **RT (min)** | **M/Z experimental** | **Teoric mass** | **MS/MS fragments** | **Molecular fornula** | **Error (ppm)** |
| **Flavanones** **[M − H] ¯** | | | | | | |
| Naringin | 17.04 | 579.1742 | 579.1719 | 2707160 | C_27_H_32_O_14_ | 3.9 |
| Narirutin | 17.04 | 579.1742 | 579.1719 | 150/270 | C_27_H_32_O_14_ | 3.9 |
| Naringenin | 18.81 | 271.0623 | 271.0612 | 116/118 | C_15_H_12_O_5_ | 4.2 |
| Chalconaringenin | 18.81 | 271.0623 | 271.0612 | 82/116/118 | C_15_H_12_O_5_ | 4.2 |
| Butein | 18.81 | 271.0623 | 271.0612 | 118 | C_15_H_12_O_5_ | 4.2 |
| Esculetin | 18.82 | 177.0195 | 177.0193 | - | C_9_H_6_O_4_ | 0.8 |
| Hesperetin | 18.87 | 301.0724 | 301.0718 | 82/106/133/163/200 | C_16_H_14_O_6_ | 2 |
| **Flavones [M − H] ¯** | | | | | | |
| Apigenin 6,8-di-C-glucoside | 16.66 | 593.1536 | 593.1512 | 254/283 | C_27_H_30_O_15_ | 4.1 |
| Luteolin 7-O-rutinoside | 16.66 | 593.1536 | 593.1512 | 254/283 | C_27_H_30_O_15_ | 4.1 |
| Luteolin 4-O-glucoside | 17.65 | 447.0939 | 447.0933 | 226/254/283 | C_21_H_20_O_11_ | 1.3 |
| Luteolin 6-C-glucoside | 17.65 | 447.0939 | 447.0933 | 226/254/283 | C_21_H_20_O_11_ | 1.3 |
| Luteolin 7-O-glucoside | 17.65 | 447.0939 | 447.0933 | 226/254/283 | C_21_H_20_O_11_ | 1.3 |
| Luteolin 8-C-glucoside | 17.65 | 447.0939 | 447.0933 | 226/254/283 | C_21_H_20_O_11_ | 1.3 |
| Hispidulin | 20.34 | 299.0563 |  | 88/118/134/184 | C_16_H_12_O_6_ | 0.6 |
| **Anthocynins [M − H] ¯** | | | | | | |
| Cyanidin 3-O-rutinoside | 16.65 | 594.1582 | 594.159 | - | C_27_H_31_O_15_ | -1.4 |
| Pelargonidin 3,5-O-diglucoside | 16.65 | 594.1582 | 594.159 | - | C_27_H_31_O_15_ | -1.4 |
| Petunidin 3-O-rutinoside | 16.63 | 624.1684 | 624.1696 | - | C_28_H_33_O_16_ | -2 |
| Petunidin 3-O-glucoside | 17.67 | 478.1095 | 478.1117 | - | C_22_H_23_O_12_ | -4.6 |
| **Stilbenes [M − H] ¯** | | | | | | |
| Trans-Resveratrol | 18.81 | 227.0718 | 227.0714 | 82/116/142 | C_14_H_12_O_3_ | 2 |
| Resveratrol | 18.81 | 227.0718 | 227.0714 | 82/116/142 | C_14_H_12_O_3_ | 2 |
| Cis-Resveratrol | 18.81 | 227.0718 | 227.0714 | 82/116/142 | C_14_H_12_O_3_ | 2 |
| **Dhydrochalcones[M − H] ¯** | | | | | | |
| Phloridzin | 17.57 | 435.1298 | 435.1297 | 122/166 | C_21_H_24_O_10_ | 0.3 |
