# Supplementary material for: Potential Use of Vacuum Impregnation and High-Pressure Homogenization to Obtain Functional Products from Lulo Fruit (Solanum quitoense Lam.)
Source: Foods. 2021 Apr 9;10(4):817. doi: 10.3390/foods10040817 (PMC8069265; doi:10.3390/foods10040817)
Supplement: Supplementary file 1 [file foods-10-00817-s001.zip › Suplementari files/Table S5. Comp. File.docx]

Table S5. Phytochemical profile of homogenized at 150 MPa lulo juice by high resolution LC-MS/MS

| **Homogenized 150 MPa** | | | | | | |
| --- | --- | --- | --- | --- | --- | --- |
| **Compound name** | **RT (min)** | **M/Z experimental** | **Teoric mass** | **MS/MS fragments** | **Molecular Formula** | **Error (ppm)** |
| **Hydroxycinnamic acids [M − H] ¯** | | | | | | |
| 3-Caffeoylquinic acid | 12.9 | 353.0875 | 353.0878 | 190/84 | C_16_H_18_O_9_ | -0.9 |
| Cis-3-Caffeoylquinic acid | 12.9 | 353.0875 | 353.0878 | 190/84 | C_16_H_18_O_9_ | -0.9 |
| Trans-5-Caffeoylquinic acid | 12.9 | 353.0875 | 353.0878 | 190/84 | C_16_H_18_O_9_ | -0.9 |
| Cis-5-Caffeoylquinic acid | 12.9 | 353.0875 | 353.0878 | 190/84 | C_16_H_18_O_9_ | -0.9 |
| 4-Caffeoylquinic acid | 12.9 | 353.0875 | 353.0878 | 190/84 | C_16_H_18_O_9_ | -0.9 |
| 5-Caffeoylquinic acid | 12.9 | 353.0875 | 353.0878 | 190/84 | C_16_H_18_O_9_ | -0.9 |
| Trans-3-Caffeoylquinic acid | 12.9 | 353.0875 | 353.0878 | 190/84 | C_16_H_18_O_9_ | -0.9 |
| 1-Caffeoylquinic acid | 12.9 | 353.0875 | 353.0878 | 190/84 | C_16_H_18_O_9_ | -0.9 |
| Caffeic acid | 13.28 | 179.0351 | 179.035 | 134/133/105/90/88 | C_9_H_8_O_4_ | 0.9 |
| Trans-Caffeic acid | 13.28 | 179.0351 | 179.035 | 134/133/105/90/88 | C_9_H_8_O_4_ | 0.9 |
| Cis-Caffeic acid | 13.28 | 179.0351 | 179.035 | 134/133/105/90/88 | C_9_H_8_O_4_ | 0.9 |
| Ferulic acid 4-O-glucoside | 13.52 | 355.1031 | 355.1035 | 174/159/131 | C_16_H_20_O_9_ | -0.9 |
| Feruloyl glucose | 13.52 | 355.1031 | 355.1035 | 174/159/131 | C_16_H_20_O_9_ | -0.9 |
| p-Coumaroylquinic acid | 14.64 | 337.0928 | 337.0929 | - | C_16_H_18_O_8_ | -0.2 |
| 3-p-Coumaroylquinic acid | 14.64 | 337.0928 | 337.0929 | - | C_16_H_18_O_8_ | -0.2 |
| 4-p-Coumaroylquinic acid | 14.64 | 337.0928 | 337.0929 | - | C_16_H_18_O_8_ | -0.2 |
| 5-p-Coumaroylquinic acid | 14.64 | 337.0928 | 337.0929 | - | C_16_H_18_O_8_ | -0.2 |
| 5-Feruloylquinic acid | 15.02 | 367.1035 | 367.1035 | 334/274/190/172/154/133/92 | C_17_H_20_O_9_ | 0.1 |
| 4-Feruloylquinic acid | 15.02 | 367.1035 | 367.1035 | 334/274/190/172/154/133/92 | C_17_H_20_O_9_ | 0.1 |
| 3-Feruloylquinic acid | 15.02 | 367.1035 | 367.1035 | 334/274/190/172/154/133/92 | C_17_H_20_O_9_ | 0.1 |
| *Table x (continued)* |  |  |  |  |  |  |
| **Compound name** | **RT (min)** | **M/Z experimental** | **Teoric mass** | **MS/MS fragments** | **Molecular fornula** | **Error (ppm)** |
| **Flavonoids [M − H] ¯** |  |  |  |  |  |  |
| Kaempferol 3,7-O-diglucoside | 15.96 | 609.1469 | 609.1461 | 270/299 | C_27_H_30_O_16_ | 1.2 |
| Quercetin 3-O-rhamnosyl-galactoside | 15.96 | 609.1469 | 609.1461 | 270/299 | C_27_H_30_O_16_ | 1.2 |
| Quercetin 3-O-galactoside 7-O-rhamnoside | 15.96 | 609.1469 | 609.1461 | 270/299 | C_27_H_30_O_16_ | 1.2 |
| Kaempferol 3-O-sophoroside | 15.96 | 609.1469 | 609.1461 | 270/299 | C_27_H_30_O_16_ | 1.2 |
| Quercetin 3-O-rutinoside | 15.8 | 609.1467 | 609.1461 | 299/270 | C_27_H_30_O_16_ | 1.0 |
| Kaempferol 3-O-galactoside | 17.48 | 447.0934 | 447.0933 | 226/254/283 | C_21_H_20_O_11_ | 0.4 |
| Kaempferol 3-O-glucoside | 17.48 | 447.0933 | 447.0934 | 226/254/283 | C_21_H_20_O_11_ | 0.4 |
| Kaempferol 7-O-glucoside | 17.48 | 447.0934 | 447.0933 | 226/254/283 | C_21_H_20_O_11_ | 0.4 |
| Quercetin 3-O-rhamnoside | 17.48 | 447.0934 | 447.0933 | 226/254/283 | C_21_H_20_O_11_ | 0.4 |
| **Phenolic acids [M − H] ¯** | | | | | | |
| 2-Hydroxybenzoic acid | 11.36 | 137.0245 | 137.0244 | 107/90 | C_7_H_6_O_3_ | 1 |
| 3-Hydroxybenzoic acid | 11.36 | 137.0245 | 137.0244 | 107/90 | C_7_H_6_O_3_ | 1 |
| 4-Hydroxybenzoic acid | 11.36 | 137.0245 | 137.0244 | 107/90 | C_7_H_6_O_3_ | 1 |
| 4-Hydroxybenzoic acid 4-O-glucoside | 12.8 | 299.0774 | 299.0772 | 136/92 | C_13_H_16_O_8_ | 0.4 |
| Benzoic acid | 14.22 | 121.0296 | 121.0295 | - | C_7_H_6_O_2_ | 0.9 |
| **Other phenolics** **[M − H] ¯** |  |  |  |  |  |  |
| Sesamol | 11.36 | 137.0245 | 137.0244 | 107/90 | C_7_H_6_O_3_ | 1 |
| 4-Hydroxycoumarin | 12.85 | 161.0245 | 161.0244 | - | C_9_H_6_O_3_ | 0.3 |
| 3,4-Dihydroxyphenyl-2-oxypropanoic acid | 13.28 | 179.0351 | 179.035 | 90/105/132/133/134 | C_9_H_8_O_4_ | 0.9 |
| 4-Hydroxybenzaldehyde | 14.22 | 121.0296 | 121.0295 | 154/144/118/116/106/92 | C_7_H_6_O_2_ | 0.9 |
| Homovanillyl alcohol | 18.27 | 167.0715 | 167.0714 |  | C_9_H_12_O_3_ | 0.6 |
| Chalconaringenin | 18.59 | 271.0614 | 271.0612 | 154/144/118/116/106/92 | C_15_H_12_O_5_ | 0.9 |
| Sitostanyl ferulate | 20.52 | 607.4364 | 607.4368 | - | C_39_H_60_O_5_ | -0.6 |
| *Table x (continued)* |  |  |  |  |  |  |
| **Compound name** | **RT (min)** | **M/Z experimental** | **Teoric mass** | **MS/MS fragments** | **Molecular fornula** | **Error (ppm)** |
| **Flavanones [M − H] ¯** | | | | | | |
| Protocatechuic aldehyde | 11.36 | 137.0245 | 137.0244 | 107/90 | C7H6O3 | 1 |
| Narirutin 4-O-glucoside | 14.19 | 741.2249 | 741.2248 | 208/193 | C_33_H_42_O_19_ | 0.2 |
| Narirutin | 16.84 | 579.1725 | 579.1719 | 270/150 | C_27_H_32_O_14_ | 0.9 |
| Naringin | 16.84 | 579.1725 | 579.1719 | 270/150 | C_27_H_32_O_14_ | 0.9 |
| Naringenin | 18.59 | 271.0614 | 271.0612 | 144/118/116/106/92 | C_15_H_12_O_5_ | 0.9 |
| Hesperetin | 16.98 | 301.0721 | 301.0718 | - | C_16_H_14_O_6_ | 1 |
| Naringenin 7-O-glucoside | 17.56 | 433.1142 | 433.114 | 270/226/186/176/150/118/106 | C_21_H_22_O_10_ | 0.4 |
| Engeletin | 17.56 | 433.1142 | 433.114 | 270/226/186/176/150/118/106 | C_21_H_22_O_10_ | 0.4 |
| Butein | 18.59 | 271.0614 | 271.0612 | 154/144/118/116/106/92 | C_15_H_12_O_5_ | 0.9 |
| **Flavones [M − H] ¯** | | | | | | |
| Luteolin 4-O-glucoside | 17.48 | 447.0934 | 447.0933 | 283/254/226 | C_21_H_20_O_11_ | 0.4 |
| Luteolin 6-C-glucoside | 17.48 | 447.0934 | 447.0933 | 283/254/226 | C_21_H_20_O_11_ | 0.4 |
| Luteolin 7-O-glucoside | 17.48 | 447.0933 | 447.0934 | 283/254/226 | C_21_H_20_O_11_ | 0.4 |
| Luteolin 8-C-glucoside | 17.48 | 447.0934 | 447.0933 | 283/254/226 | C_21_H_20_O_11_ | 0.4 |
| 6-Hydroxyluteolin 7-O-rhamnoside | 17.48 | 447.0934 | 447.0933 | 283/254/226 | C_21_H_20_O_11_ | 0.4 |
| **Flavonols [M − H] ¯** |  |  |  |  |  |  |
| 5,4-Dihydroxy-3,3-dimethoxy-6:7-methylenedioxyflavone | 19.26 | 357.062 | 357.0616 | 182/179/120/125/112/120 | C_18_H_14_O_8_ | 1.1 |
| **Anthocynins [M − H] ¯** | | | | | | |
| Malvidin 3-O-(6-acetyl-galactoside | 2.68 | 534.1387 | 534.1379 | 190/110/86 | C_25_H_27_O_13_ | 1.5 |
| Malvidin 3-O-(6-acetyl-glucoside) | 2.68 | 534.1387 | 534.1379 | 190/110/86 | C_25_H_27_O_13_ | 1.5 |
| Cyanidin 3-O-laminaribioside | 15.96 | 610.1516 | 610.1539 | 300/271 | C_27_H_31_O_16_ | -3.8 |
| Cyanidin 3-O-sophoroside | 15.96 | 610.1539 | 610.1516 | 300/271 | C_27_H_31_O_16_ | -3.8 |
| Delphinidin 3-O-rutinoside | 15.96 | 610.1516 | 610.1539 | 300/271 | C_27_H_31_O_16_ | -3.8 |
| **Dhydrochalcones** **[M − H] ¯** |  |  |  |  |  |  |
| Phloridzin | 17.39 | 435.1294 | 435.1297 | 272/254/178/166/150/188 | C_21_H_24_O_10_ | -0.2 |
